# Spark Plasma Sintering of WC-Based 10wt%Co Hard Alloy: A Study of Sintering Kinetics and Solid-Phase Processes

**DOI:** 10.3390/ma15031091

**Published:** 2022-01-30

**Authors:** Anastasia A. Buravleva, Alexander N. Fedorets, Anastasia A. Vornovskikh, Alexey V. Ognev, Valeria A. Nepomnyushchaya, Vladimir N. Sakhnevich, Aleksey O. Lembikov, Zlata E. Kornakova, Olesya V. Kapustina, Anna E. Tarabanova, Victor P. Reva, Igor Yu. Buravlev

**Affiliations:** 1 Far Eastern Federal University, 10 Ajax Bay, Russky Island, 690922 Vladivostok, Russia; bouy@mail.ru (A.A.B.); fedorec.an@dvfu.ru (A.N.F.); vornovskikh_aa@students.dvfu.ru (A.A.V.); vnepomnyushchaya@mail.ru (V.A.N.); sakhnevich.vn@students.dvfu.ru (V.N.S.); lembikov.ao@students.dvfu.ru (A.O.L.); kornakova.ze@students.dvfu.ru (Z.E.K.); kapusto.2001@mail.ru (O.V.K.); anna.t108@yandex.ru (A.E.T.); reva.vp@dvfu.ru (V.P.R.); 2 Laboratory of Spin-Orbitronics, Institute of High Technologies and Advanced Materials, Far Eastern Federal University, 690922 Vladivostok, Russia; ognev.av@dvfu.ru

**Keywords:** tungsten carbide (WC), hard metal alloy, WC-Co, spark plasma sintering (SPS), grain size

## Abstract

The paper describes the method for producing WC-10wt%Co hard alloy with 99.6% of the theoretical density and a Vickers hardness of ~1400 HV 0.5. Experimental data on densification dynamics, phase composition, morphology, mechanical properties, and grain size distribution of WC-10%wtCo using spark plasma sintering (SPS) within the range of 1000–1200 °C are presented. The high quality of the product is provided by the advanced method of high-speed powder mixture SPS-consolidation at achieving a high degree of densification with minimal calculated grain growth at 1200 °C.

## 1. Introduction

Tungsten carbide (WC)-based hard alloys represent an important class of materials for various engineering applications [1,2,3,4,5,6,7,8]. Today, WC can be considered the most popular material, which is most widely used in industry to produce a wide range of tool cutting products. Due to such properties as high hardness (80–92 HRA) and heat resistance (800–1000 °C), hard alloys can be used at high cutting speeds. Due to the high demand and widespread use, hard alloys based on various binders continue to be studied intensively, as evidenced by a large number of publications in recent years. It is applied to the use of WC in composite coatings [9], coatings on high-strength steel [10], and coatings for tribotechnical purposes [11]. Furthermore, the use of WC is actively studied for the tasks of diamond tool making [12], as one of the components of the filler of matrices of high hardness materials based on the diamond [13] and in the composition of the diamond film [14].

Among the relevant problems traditionally solved by current science and industry are increasing the strength of hard alloys and compensating the drawbacks associated with the lack of impact toughness. Furthermore, much attention is paid to studying the effect of thermal cycling modes on hard alloys’ mechanical and tribological properties. The processes of WC material fabrication by electro spark deposition (ESD) and laser beam machining (LBM) methods and also Cr-WC electrodeposition by direct (DC) and pulsed (PC) currents are topical [15,16]. WC production simplification and cheapening tasks deserve particular attention [17]. Furthermore, the issues of recycling and utilization of WC-containing materials do not lose their relevance [18].

The high sintering temperature of WC causes the use of metal-bonding alloys to synthesize WC, which gives the products bending strength. Research is continuing in the direction of studying the use of traditional and alternative binders based on the combination of iron and aluminum [19], Co and Ni_3_Al [20], Fe/Ni [21], as well as alternative binders based on metals with high melting temperatures accompanied by a phase formation during spark plasma sintering (SPS) reaction [22]. There are known cases of the application of complex medium-entropic compositions of hardened combined binders, including those used to manufacture alloys of high-tech products such as gas turbines [23]. Chromium-based transition metal borides (CrB_2_ [24]) are used to manufacture metal-cutting tools suitable for work in extreme conditions. WC is used as a cermet component with multi-component composition [25]. However, despite the widely conducted research with alternative binders, the most popular alloy is still based on the WC-Co system. Around 90% of tungsten carbide hard metals are made using a cobalt binder [26,27,28]. The possibility of obtaining composite WC-Co system micro-powders with spherical particle shape having submicron and nanoscale (50 nm) structure [29] with uniform distribution of W, Co, and C among powder nanoparticles in the nanoscale range has been widely experimentally confirmed. Recent studies indicate the possibility of obtaining ultrafine spherical microparticles by electric discharge erosion of cemented WC-Co [30], also in DC arc discharge plasma [31].

Combining the sintering process with pressing pressure is justified for the formation of high-density WC structures of hard alloys. In particular, SPS belongs to perspective and well-proven methods of obtaining products from powdered materials, including WC hard alloys [21,32,33,34,35,36]. SPS demonstrates clear advantages over conventional consolidation methods of powder compositions, including hot isostatic pressing (HIP) and sintering, high-frequency induction heating, isostatic pressing and sintering. Significant compaction is achieved at a relatively lower temperature and shorter time [21,36,37,38,39,40,41,42,43,44,45,46,47,48,49,50,51,52,53]. This process provides a high rate of heating and sintering of a dense product in one stage and, in some cases, avoids the introduction of carbide grain growth inhibitors into the consolidated reaction mixture. For WC alloys, the SPS method, compared to traditional sintering methods, demonstrates the smallest grain size, the highest hardness, the lowest fracture toughness [54]. The application of the SPS technology makes it possible to reduce the sintering temperatures by 200 °C, which preserves the WC grain size, uses alternative molds [55], and provides reactive synthesis [56]. Considerable experience has been accumulated in using traditional and modern methods of sintering WC-Co nanopowders produced by plasma chemical synthesis [57]. The influence of carbon on the SPS kinetics of WC-10wt%Co nano- and submicron powders has been investigated [58]. Publications of recent years show that the technology of alloy production of this standard system is developing, in particular, by microwave synthesis [59]. Hard alloys produced by SPS [60] and a combination of mechanochemistry and SPS [61] are being synthesized. SPS has proven to be a perfect method for consolidation of various applications [36,55,62,63,64], porosity reduction for materials where high density is a critical property [65], including hard alloys and high-temperature materials [66,67,68], and coating [69,70,71]. The issue of controlling the grain growth of ceramic materials during sintering in various media remains relevant [50,72,73,74]. It is widely known that SPS preserves the grain size of a wide range of ceramic materials for various purposes due to rapid heating at constant pressure [75,76,77].

The purpose of the present paper was to study the dilatometry features of 10 wt% cobalt binder WC-based hard alloy SPS synthesis, with further study of phase composition, microstructure (grain size), and physical-mechanical properties. The composition of WC -based hard alloy with 10 wt% cobalt binder is chosen due to the widespread use of this composition of tungsten-cobalt single-carbide alloys in the industry as a material for manufacturing cutting tools, wear parts of machines and devices, for making tools and accessories for material pressure treatment processes, for making precise measuring tools, etc.

## 2. Materials and Methods

### 2.1. Materials

WC, Co commercial powders with a mass fraction of 99.9% (Sigma-Aldrich) were used to synthesize the initial mixtures.

### 2.2. Initial Powder Production

Preparing of WC-10%Co initial mixtures was carried out using Changsha Tianchuang Powder Technology Co. Ltd. (Changsha, China) tungsten carbide grinding bowls on vertical planetary ball mill Tencan XQM-0.4A (Changsha, China). WC-balls with diameters of 10 mm and 5 mm were used as grinding bodies. A total of 50 g of powder (WC—45 g; Co—5 g) was milled in the presence of 15 balls with a total mass of 125 g. Milling was carried out at 700 rpm for 7 cycles; each cycle consisted of 15 min of milling followed by 15 min of cooling of the milling vessel. Grinding was carried out wet in an anhydrous isopropanol medium (50 mL). To prevent powder materials oxidation, isopropanol was pretreated with argon to remove dissolved oxygen, and argon was additionally pumped into the milling container to displace air. After grinding was completed, the container was depressurized and placed in an oven at 80 °C to remove the isopropanol.

### 2.3. SPS Consolidation

Spark plasma sintering of the samples was carried out on an SPS-515S machine (Dr. Sinter LAB^TM^, Tokyo, Japan) at a constant pressing pressure of 57.3 MPa and heating rate of 85–90 °C/min. The maximum sintering temperature did not exceed 1200 °C. Sintering was carried out according to the following scheme: 8 g of powder was placed in a graphite mold (diameter of 10.5 mm), then pressed (20 MPa) and placed in the sintering chamber, vacuumed (10^–5^ atm), and where were sintered. The temperature of the SPS process was controlled by an optical pyrometer Hitachi IR-AHS (Tokyo, Japan).

### 2.4. Characteristics of Research Methods

The powders’ particle size distribution (PSD) was determined on a laser particle analyzer Analysette-22 NanoTec/MicroTec/XT (Fritsch, Germany). Each sample was measured three times; then, the results were averaged.

Hard alloys samples were examined from a pre-polished cross-sectional surface. The samples were sawed with a diamond disk at a rotational speed of 3000 rpm while cooling with a water emulsion solution on an automatic high-speed precision cutting machine Metkon Micracut 201 at a cutting speed of 2 mm/min.

Automatic polishing of samples following the standards of preparing samples for microstructure studies was carried out on an automatic grinding and polishing station PRESI MECATECH 234 (Grenoble, France).

X-ray diffraction analysis (XRD) of the initial powders and alloys was carried out on a multipurpose X-ray diffractometer D8 Advance Bruker AXS (Karlsruhe, Germany). CuKα radiation, Ni filter, average wavelength (λ) 1.5418 Å, shooting angle range 10–80°, scanning step 0.02°, recording rate of spectra—5°/min. The scanning electron microscopy images were captured using an ULTRA 55+ (ZEISS, Jena, Germany) scanning electron microscope (SEM) operating at 2 kV, which was also used to record the elemental mapping with an X-Max 80 EDS system (Oxford Instruments, Abingdon, UK) at 20kV. Grain size distribution and average grain size of the samples were calculated by the linear intercept method [78,79]. At least 300 grains were analyzed for each measurement.

The specific gravity of sintered samples was measured by hydrostatic weighing on AdventurerTM OHAUS Corporation (Parsippany, NJ, USA) scales. Calculation of the relative density from the theoretical alloy samples was carried out by the formula (RD):

(1) $$\mathrm{RD}\left(\%\right)=\frac{100}{\frac{\omega_{1}}{\rho_{1}}+\frac{\omega_{2}}{\rho_{2}}}$$

where, *ω*—mass fraction of the component, *ρ*—theoretical density of the component.

Microhardness of the samples obtained was measured at a load of HV0.5 (4.903 N) on a microhardness tester Shimadzu HMV-G-FA-D (Kyoto, Japan).

## 3. Results and Discussion

### 3.1. WC Grinding

**Figure 1:**
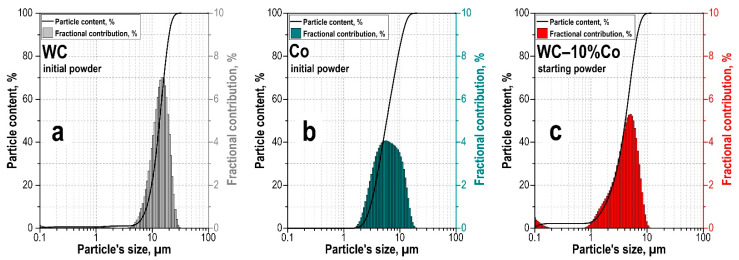
Particle size distribution of the initial (**a**) WC and (**b**) Co powders, as well as (**c**) after grinding the mixture WC-10wt%Co for 7 grinding cycles.

The particle size distribution of WC-10%Co powder after seven milling cycles obtained from the initial raw materials has particle size WC 4–30 μm, Co particles 2–20 μm, represented by a broad fraction of particles 0.1–10 μm in size (Figure 1). The minor fraction up to 100 nm represents less than 4% of the total powder. The main fraction is represented by particles lying in the 1–10 μm size range and making up more than 95% of all particles. It is shown that in this milling mode, the WC particle size decreases by 30% of the original size.

### 3.2. SPS Consolidation of Powder of the WC-10Co System

The sintering temperature range of 1000–1200 °C is chosen for several reasons. The temperature value is limited by the melting temperature of the cobalt binder. The analysis of the dilatometric curves shows that exceeding the temperature above 1200 °C leads to excessive melting and leakage of the cobalt binder in a sample. At the same time, the sintering temperature of WC alloys by the SPS method is significantly lower (200–250 °C) than in the case of traditional liquid-phase sintering methods (1400–1500 °C) due to the specifics of powder sintering in a spark plasma current with simultaneous application of external pressing pressure.

The kinetics of WC-10wt%Co powder shrinkage while sintering with pressure assisting (57.3 MPa) was studied by consolidation dynamics under heating to 1200 °C. The temperature measurement started at 110 s of the sintering process (counting at ~580 °C).

**Figure 2:**
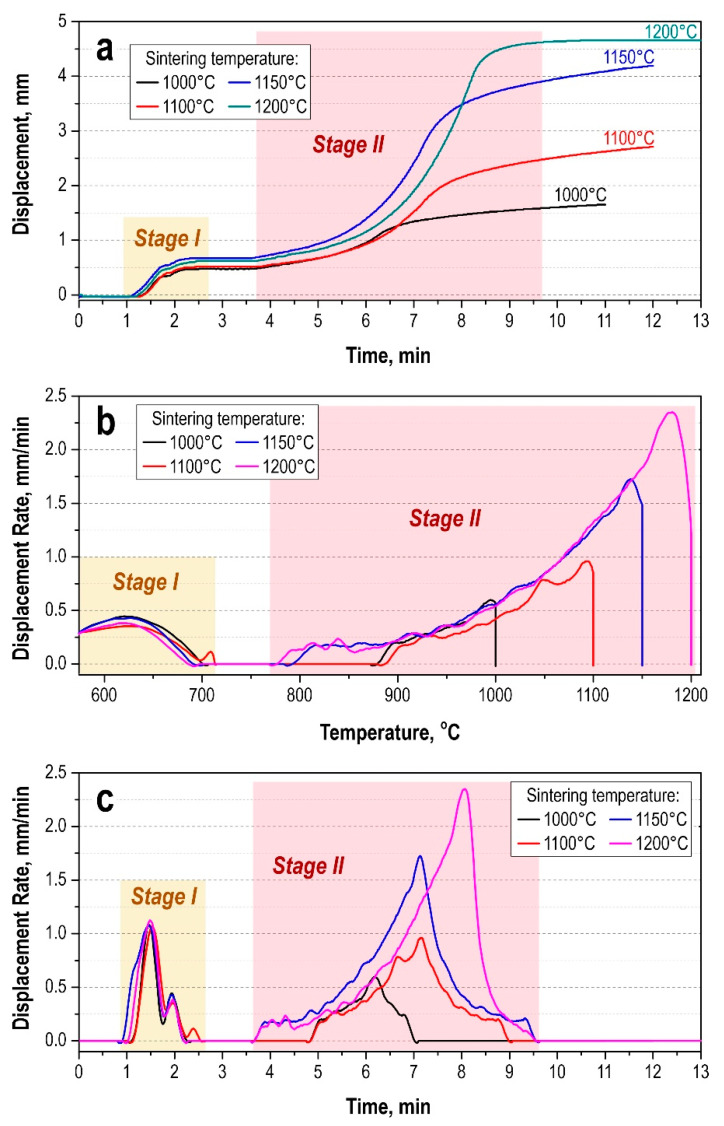
Compaction of WC-10wt%Co powder with different SPS temperatures (1000, 1100, 1150, 1200 °C): (**a**) displacement/time; (**b**) displacement rate/temperature; (**c**) displacement rate/time.

Analysis of the shrinkage rate time-dependence curves (Figure 2) made it possible to establish that compaction of the powder understudy at the initial stage had the same character for all samples. During the first two minutes of the sintering process, the powder is compacted due to mechanical impact, consisting of the destruction and regrouping of WC particles.

The beginning of intensive densification and sintering of the powder for all samples occurs at 780 ± 2 °C and passes to the active stage in the range of 900–1060 °C at 5–6.5 min of the process. Dynamic compaction during heating in this range is related to the intensification of plastic and creeping currents in the sintered powder, and the initial temperature of the active sintering stage depends on the heating rate. The shrinkage of the sample is slowing down after the cessation of heating.

**Figure 3:**
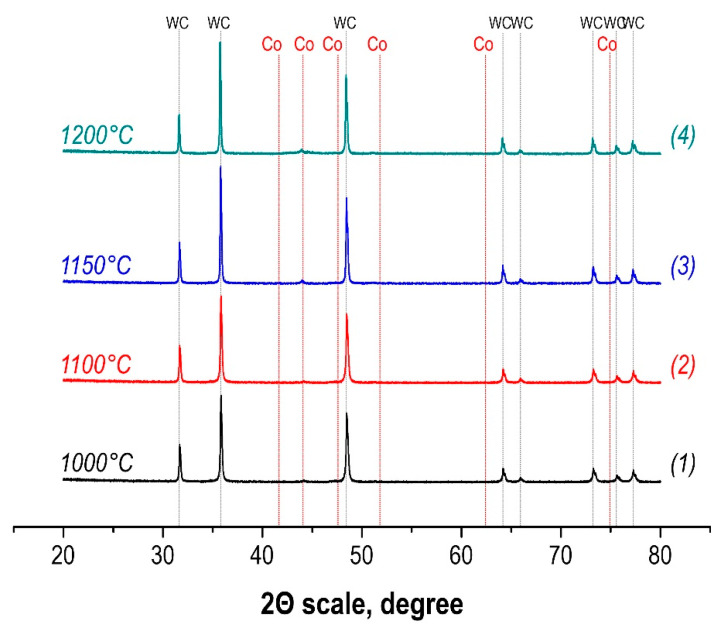
Phase composition of WC-10wt%Co hard alloys obtained by SPS-consolidation at different temperatures (1000, 1100, 1150, 1200 °C).

A marked increase in the density of the samples occurs with an increase in the sintering temperature in the value range from 1000 °C to 1150 °C. At the same time, significant densification of the powder mixture during sintering occurs in the range of 1150–1200 °C (Figure 2) with reaching the densest packing at 1200 °C without recording further densification. The invariance of the phase composition of the SPS-consolidated samples over the entire range of temperature values is confirmed by the XRD results (Figure 3). With increasing sintering temperature, the distribution of the cobalt binder in the matrix improves with increasing homogeneity of the alloy.

**Figure 4:**
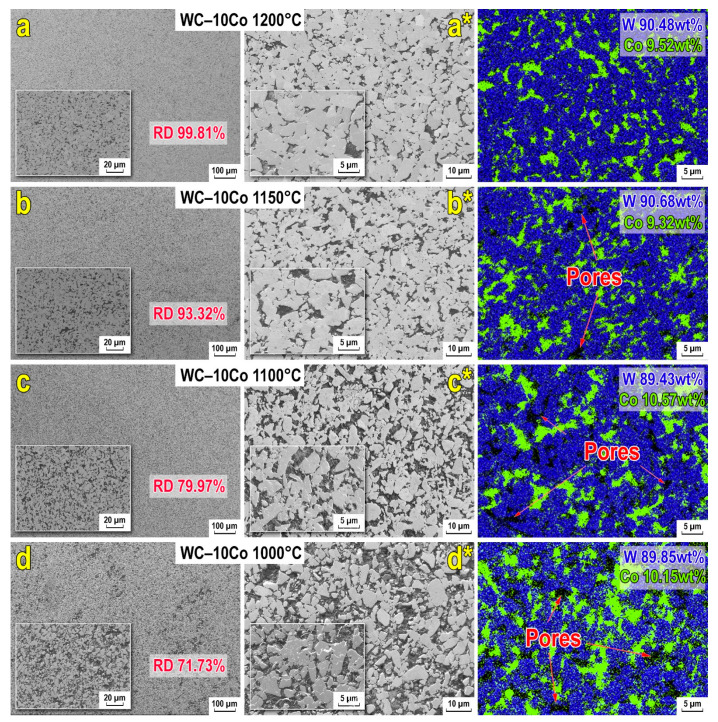
Microstructure and EDS maps of WC-10wt%Co hard alloys obtained via SPS-consolidation at various sintering temperatures: (**a**,a*) 1200 °C; (**b**,b*) 1100 °C; (**c**,c*) 1150 °C; (**d**,d*) 1000 °C (RD—relative density).

Figure 4 shows the secondary electron images (SEI) of the cross-sectional surface of a sintered WC-10wt%Co samples indicate a significant influence of the sintering temperature during consolidation on the packing density of WC and especially on the quality of the cobalt binder distribution in the sample volume. The lowest of the selected sintering temperatures (1000 °C) leads to insufficient compaction of the sintered mixture, as evidenced by the residual structure defects in the form of significant pores and voids (up to several microns in size). At the same time, the insufficient cobalt binder’s ability to spill in the sample limits its entry into the dead-end areas between the WC grains, formed due to their direct contact. At the same time, areas of increased concentration of the cobalt binder in the WC volume are observed (Figure 4). Sintering in the temperature range of 1100–1200 °C as a result of the intensification of the liquid-phase sintering process densification occurs due to improved flowability of the Co binder, which results in a qualitative change of the alloy microstructure, due to reduction of the number of pores and increases in density (Figure 4) with achieving the best distribution indicators of the cobalt binder at sintering at 1200 °C (Figure 4a,a*).

**Figure 5:**
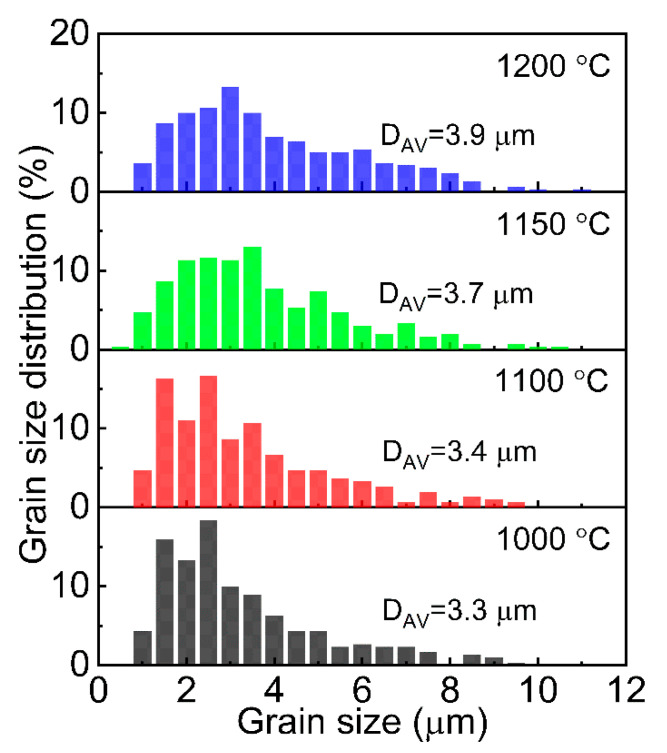
Grain size distribution of WC in WC-10wt%Co alloys SPSed at 1000–1200 °C.

Analysis of the morphology of WC-10wt%Co alloys indicates that with sintering temperature increasing from 1000 up to 1200 °C, the average size of grains slightly increases from 3.3 μm to 3.9 μm with the contribution of the more significant grain fraction with size > 4 μm. However, the maximum size does not exceed ~11 µm (Figure 5). The result obtained is related to the fact that thermally activated mass transfer processes aim to decrease the system energy by reducing the intergranular boundaries [80]. In this case, the growth of WC grains is limited by the number and branching of the Co-bonding channel networks in the WC volume (Figure 4). In this connection, the additional separation by increasing the fluid flowability of the Co phase made it possible to avoid the uncontrolled growth of WC grains even at relatively high SPS temperatures (in the context of our WC-10wt%Co system).

In conventional sintering of WC-Co powder compositions, the sintering temperature is above the eutectic temperature of WC-Co. The temperature of 1200 °C is the temperature generated directly by the automation of the SPS unit and is additionally measured from the surface of the graphite die using an optical pyrometer. The temperature determined at the survey point from the surface of the mold (calculated temperature) and the local temperature in the microregions on the surface of the material grains differs significantly due to the specifics of the SPS process. The primary sintering energy (Joule–Lenz heat) is generated at the grain boundary. At these contact points, the material in the near-surface region can almost instantaneously transition to a plasma state. These are very small microregions, and their number in the entire sample volume can be significant. However, this does not mean that this effect is observed for every particle and in every contact. It is impossible to measure the temperature in such microregions, so the total temperature of the system is measured after the heat from the microregions has been distributed and equilibrated throughout the sample. We can state an excess of the actual local temperature in the sintering zone for the cobalt binder based on the results in paper [81], which presents a physical model according to which the actual cobalt melting temperature exceeds the design sintering temperature of the SPS process during sintering. Consequently, the temperature in the sintering region can indeed exceed the eutectic temperature in the microvolume (at the particle boundary). Thus, it is fair to state that since the maximum sintering temperature for a given alloy composition is limited by the melting temperature of the cobalt bond, the maximum grain growth in this sintering temperature range is also limited by the melting temperature conditions of cobalt.

**Figure 6:**
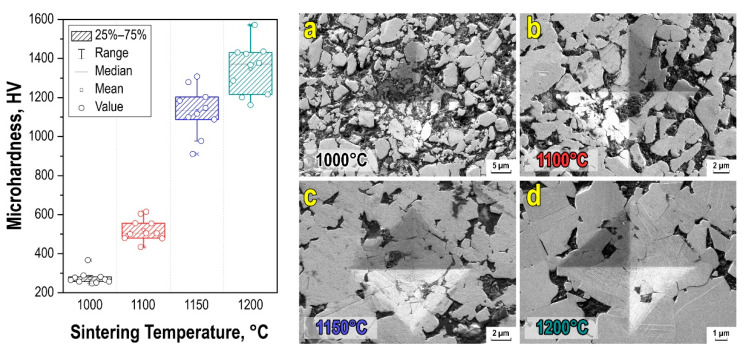
Vickers microhardness dispersion (**left**) of WC-10wt%Co ceramic samples, and microphotographs of microhardness indentor prints at HV0.5 on the surface of tested samples (**right**) WC-10wt%Co obtained by SPS consolidation at different temperatures: (**a**)—1000 °C (×5000); (**b**)—1100 °C (×10,000); (**c**)—1150 °C (×12,000); (**d**)—1200 °C (×15,000).

The surface images with indentation of the micro-hardness tester at HV0.5 show the absence of cracking tendency in SPS-derived WC-10wt%Co hard alloys at various sintering temperatures (Figure 6). The microhardness of the WC-10wt%Co hard alloy obtained via SPS at the optimal sintering temperature (1200 °C) is comparable with commercial WC-Co medium grain hard alloys. The primary mechanism of the alloy components compaction is the rearrangement of tungsten carbide particles, the enhancement of diffusion processes, and the viscous flow of the cobalt binder.

To analyze the microhardness, a “box-and-whiskers” diagram was plotted, which is an indirect estimate of the strength microheterogeneity of the material (Figure 6). The data obtained show that the hardness of materials grows from ~300 to 1600 HV with increasing sintering temperature. The non-uniform scatter of hardness values can be compared with the grain growth. For example, up to 1100 °C “box with whiskers” the scatter of values decreases and corresponds to 500 HV, and when the temperature increases up to 1200 °C, along with the formation of WC-Co monophase active growth of agglomerates occurs as revealed by SEM (Figure 4), which results in anisotropy of characteristics in local areas of material volume.

## 4. Conclusions

The practical method of WC-10wt%Co alloy production by SPS consolidation was demonstrated. It was experimentally proved that SPS provides WC-10wt%Co alloy at 1000, 1100, 1150, and 1200 °C with short heating and holding time, not more than 13 min. Optimal parameters of SPS consolidation (1200 °C, 57.3 MPa, 10 min) of WC-based hard alloy containing Co binder for binder spreading in the alloy volume have been determined. Physical and mechanical studies have established that the hardness and mechanical strength of WC-10wt%Co alloy samples increase up to the sintering temperature. The sample reaches 99.6% density of the theoretical value, hardness ~1400 HV 0.5 without cracking. Analysis of the WC-10wt%Co hard alloys morphology indicates that with increasing temperature from 1000 to 1200 °C, the average WC grain size increases slightly from 3.3 μm to 3.9 μm with an increasing contribution of a fraction of larger grains with a size > 4 μm.

## References

[1] Ettmayer P., Kolaska H., Ortner H.M. (2014). History of hardmetals. Comprehensive Hard Materials.

[2] Gee M.G., Gant A., Roebuck B. (2007). Wear mechanisms in abrasion and erosion of WC/Co and related hardmetals. Wear.

[3] Rizzo A., Goel S., Grilli M.L., Iglesias R., Jaworska L., Lapkovskis V., Novak P., Postolnyi B.O., Valerini D. (2020). The critical raw materials in cutting tools for machining applications: A review. Materials.

[4] Humphry-Baker S.A., Ramanujam P., Smith G.D.W., Binner J., Lee W.E. (2020). Ablation resistance of tungsten carbide cermets under extreme conditions. Int. J. Refract. Met. Hard Mater..

[5] Humphry-Baker S.A., Harrison R.W., Greaves G., Knowles A.J., Smith G.D.W., Donnelly S.E., Lee W.E. (2018). A candidate fusion engineering material, WC-FeCr. Scr. Mater..

[6] Pittari J.J., Murdoch H.A., Kilczewski S.M., Hornbuckle B.C., Swab J.J., Darling K.A., Wright J.C., Materials H., Pittari J.J., Murdoch H.A. (2018). Sintering of tungsten carbide cermets with an iron-based ternary alloy binder: Processing and thermodynamic considerations. Int. J. Refract. Met. Hard Mater..

[7] Windsor C.G., Morgan J.G., Buxton P.F., Costley A.E., Smith G.D.W., Sykes A. (2017). Modelling the power deposition into a spherical tokamak fusion power plant. Nucl. Fusion.

[8] Windsor C.G., Marshall J.M., Morgan J.G., Fair J., Smith G.D.W., Rajczyk-Wryk A., Tarragó J.M. (2018). Design of cemented tungsten carbide and boride-containing shields for a fusion power plant. Nucl. Fusion.

[9] Krylova T.A., Chumakov Y.A., Vasilyeva M.P. (2022). Microstructure and properties of WC-Ni3Al composite coatings fabricated by non-vacuum electron beam cladding. Mater. Lett..

[10] Hasan M., Zhao J., Huang Z., Wu H., Jia F., Jiang Z. (2019). Effects of holding time on the sintering of cemented tungsten carbide powder and bonding with high-strength steel wire. J. Mater. Eng. Perform..

[11] Wang W., Wang Z., Li Y., Wang D., Li M., Chen Q. (2019). Wear behavior of Fe-WC/metal double layer coatings fabricated by resistance seamweld method. Jinshu Xuebao Acta Metall. Sin..

[12] Kozyrev E.N., Kumykov V.K., Kushhabiev A.S., Manukyants A.R., Kasumov Y.N., Sozaev V.A. (2020). Development of diamond–metal compositions for diamond tools. J. Surf. Investig. X-ray Synchrotron Neutron Tech..

[13] Sharin P.P., Akimova M.P., Yakovleva S.P., Popov V.I. (2021). The structure and microhardness of binding for diamond tools based on tungsten carbide obtained by impregnation with an iron–carbon melt. Inorg. Mater. Appl. Res..

[14] Vokhmyanin D.S., Oglezneva S.A. (2019). Growth features of diamond films on the tungsten carbide surface with a copper sublayer. Russ. J. Non-Ferrous Met..

[15] Radek N., Pietraszek J., Gądek-Moszczak A., Orman Ł.J., Szczotok A. (2020). The morphology and mechanical properties of ESD coatings before and after laser beam machining. Materials.

[16] Yadlapalli B.K., Maharana H.S., Basu A. (2020). Structure and properties of pulse electrodeposited Cr–WC coating. Surf. Topogr. Metrol. Prop..

[17] Pak A.Y., Shanenkov I.I., Mamontov G.Y., Kokorina A.I. (2020). Vacuumless synthesis of tungsten carbide in a self-shielding atmospheric plasma of DC arc discharge. Int. J. Refract. Met. Hard Mater..

[18] Kamimoto Y., Kasuga R., Takeshita K., Hagio T., Kuroda K., Ichino R., Deevanhxay P. (2020). Electrochemical behavior of tungsten carbide-cobalt alloy using molten hydroxide as electrolyte under low temperature. J. Mater. Cycles Waste Manag..

[19] Mostajeran A., Shoja-Razavi R., Hadi M., Erfanmanesh M., Barekat M., Savaghebi Firouzabadi M. (2020). Evaluation of the mechanical properties of WC-FeAl composite coating fabricated by laser cladding method. Int. J. Refract. Met. Hard Mater..

[20] Peng Y., Li T., Long J., Li H., Lu B., Chen F., Du Y. (2021). Effect of bimodal WC particle size and binder composition on the morphology of WC grains in WC–Co–Ni3Al cemented carbides. J. Mater. Res. Technol..

[21] Shichalin O.O., Buravlev I.Y., Portnyagin A.S., Dvornik M.I., Mikhailenko E.A., Golub A.V., Zakharenko A.M., Sukhorada A.E., Talskikh K.Y., Buravleva A.A. (2020). SPS hard metal alloy WC-8Ni-8Fe fabrication based on mechanochemical synthetic tungsten carbide powder. J. Alloys Compd..

[22] Shichalin O.O., Buravlev I.Y., Papynov E.K., Golub A.V., Belov A.A., Buravleva A.A., Sakhnevich V.N., Dvornik M.I., Vlasova N.M., Gerasimenko A.V. (2022). Comparative study of WC-based hard alloys fabrication via spark plasma sintering using Co, Fe, Ni, Cr, and Ti binders. Int. J. Refract. Met. Hard Mater..

[23] Edtmaier C., Wolf M., de Oro Calderon R., Schubert W.-D. (2021). Effect of nickel on the formation of γ/γ′ microstructures in WC/Co-Ni–Al–W. Int. J. Refract. Met. Hard Mater..

[24] Ratov B.T., Bondarenko M.O., Mechnik V.A., Strelchuk V.V., Prikhna T.A., Kolodnitskyi V.M., Nikolenko A.S., Lytvyn P.M., Danylenko I.M., Moshchil V.E. (2021). Structure and properties of WC–Co composites with different CrB2 concentrations, sintered by vacuum hot pressing, for drill bits. J. Superhard Mater..

[25] Wu H., Zheng Y., Zhang J., Ke Z., Xu X. (2021). Influence of WC content on microstructure and mechanical properties of Mo_2_FeB_2_-based cermets fabricated by multi-step sintering. Ceram. Int..

[26] Fernandes C.M., Senos A.M.R. (2011). Cemented carbide phase diagrams: A review. Int. J. Refract. Met. Hard Mater..

[27] Kim H.C., Shon I.J., Yoon J.K., Doh J.M., Munir Z.A. (2006). Rapid sintering of ultrafine WC–Ni cermets. Int. J. Refract. Met. Hard Mater..

[28] Reva V.P., Onishchenko D.V., Petrov V.V., Kim V.A., Evstigneev A.I. (2013). Formation of hard alloy VK8 using tungsten carbide powder synthesized by mechanochemical technology. Refract. Ind. Ceram..

[29] Samokhin A., Alekseev N., Astashov A., Dorofeev A., Fadeev A., Sinayskiy M., Kalashnikov Y. (2021). Preparation of W–C–Co composite micropowder with spherical shaped particles using plasma technologies. Materials.

[30] Dvornik M., Mikhailenko E., Nikolenko S., Vlasova N., Skiruta A. (2020). Production of ultrafine-grained spherical β-WC–W_2_C–Co microparticles by electro discharge erosion of WC–15Co alloy in glycerol and their solutions. Mater. Res. Express.

[31] Pak A.Y., Kokorina A.I. (2021). Effect of energy on the phase composition of the product of arc discharge synthesis in the tungsten–carbon system obtained in a self-shielding autonomous gas environment. Inorg. Mater. Appl. Res..

[32] Raihanuzzaman R.M., Rosinski M., Xie Z., Ghomashchi R. (2016). Microstructure and mechanical properties and of pulse plasma compacted WC–Co. Int. J. Refract. Met. Hard Mater..

[33] SIWAK P., GARBIEC D. (2016). Microstructure and mechanical properties of WC–Co, WC–Co–Cr3C2 and WC–Co–TaC cermets fabricated by spark plasma sintering. Trans. Nonferrous Met. Soc. China.

[34] Liu K., Wang Z., Yin Z., Cao L., Yuan J. (2018). Effect of Co content on microstructure and mechanical properties of ultrafine grained WC–Co cemented carbide sintered by spark plasma sintering. Ceram. Int..

[35] Munir Z.A., Ohyanagi M. (2021). Perspectives on the spark plasma sintering process. J. Mater. Sci..

[36] Papynov E.K., Shichalin O.O., Medkov M.A., Grishchenko D.N., Tkachenko I.A., Fedorets A.N., Pechnikov V.S., Golub A.V., Buravlev I.Y., Tananaev I.G. (2018). Spark plasma sintering of special-purpose functional ceramics based on UO_2_, ZrO_2_, Fe_3_O_4_/α-Fe_2_O_3_. Glas. Phys. Chem..

[37] Rong H., Peng Z., Ren X., Peng Y., Wang C., Fu Z., Qi L., Miao H. (2012). Ultrafine WC–Ni cemented carbides fabricated by spark plasma sintering. Mater. Sci. Eng. A.

[38] Guillon O., Gonzalez-Julian J., Dargatz B., Kessel T., Schierning G., Räthel J., Herrmann M. (2014). Field-assisted sintering technology/spark plasma sintering: Mechanisms, materials, and technology developments. Adv. Eng..

[39] Morsi K. (2017). Combustion synthesis and the electric field: A review. Int. J. Self-Propagating High-Temp. Synth..

[40] Mamedov V. (2002). Spark plasma sintering as advanced PM sintering method. Powder Metall..

[41] Dvornik M.I., Zaitsev A.V. (2018). Variation in strength, hardness, and fracture toughness in transition from medium-grained to ultrafine hard alloy. Russ. J. Non-Ferrous Met..

[42] Pan Y., Liu A., Huang L., Du Y., Jin Y., Yang X., Zhang J. (2019). Effects of metal binder content and carbide grain size on the microstructure and properties of SPS manufactured WC–Fe composites. J. Alloys Compd..

[43] Chuvil’deev V.N., Blagoveshchenskiy Y.V., Nokhrin A.V., Sakharov N.V., Boldin M.S., Isaeva N.V., Shotin S.V., Lopatin Y.G., Smirnova E.S., Popov A.A. (2015). Sparking plasma sintering of tungsten carbide nanopowders. Nanotechnol. Russ..

[44] Chuvil’deev V.N., Blagoveshchenskii Y.V., Boldin M.S., Moskvicheva A.V., Sakharov N.V., Nokhrin A.V., Isaeva N.V., Shotin S.V., Lopatin Y.G., Pisklov A.V. (2014). High-speed electropulse plasma sintering of nanostructured tungsten carbide: Part 1. Experiment. Russ. J. Non-Ferrous Met..

[45] Chuvil’deev V.N., Blagoveshchenskiy Y.V., Nokhrin A.V., Boldin M.S., Sakharov N.V., Isaeva N.V., Shotin S.V., Belkin O.A., Popov A.A., Smirnova E.S. (2017). Spark plasma sintering of tungsten carbide nanopowders obtained through DC arc plasma synthesis. J. Alloys Compd..

[46] Biesuz M., Grasso S., Sglavo V.M. (2020). What’s new in ceramics sintering? A short report on the latest trends and future prospects. Curr. Opin. Solid State Mater. Sci..

[47] Ghasali E., Alizadeh M., Niazmand M., Ebadzadeh T. (2017). Fabrication of magnesium-boron carbide metal matrix composite by powder metallurgy route: Comparison between microwave and spark plasma sintering. J. Alloys Compd..

[48] Munir Z.A., Anselmi-Tamburini U., Ohyanagi M. (2006). The effect of electric field and pressure on the synthesis and consolidation of materials: A review of the spark plasma sintering method. J. Mater. Sci..

[49] Papynov E.K., Shichalin O.O., Mayorov V.Y., Modin E.B., Portnyagin A.S., Tkachenko I.A., Belov A.A., Gridasova E.A., Tananaev I.G., Avramenko V.A. (2017). Spark plasma sintering as a high-tech approach in a new generation of synthesis of nanostructured functional ceramics. Nanotechnol. Russ..

[50] Simonenko T.L., Kalinina M.V., Simonenko N.P., Simonenko E.P., Glumov O.V., Mel’nikova N.A., Murin I.V., Shichalin O.O., Papynov E.K., Shilova O.A. (2018). Spark plasma sintering of nanopowders in the CeO_2_-Y_2_O_3_ system as a promising approach to the creation of nanocrystalline intermediate-temperature solid electrolytes. Ceram. Int..

[51] Chuvil’deev V.N., Blagoveshchenskii Y.V., Sakharov N.V., Boldin M.S., Nokhrin A.V., Isaeva N.V., Shotin S.V., Lopatin Y.G., Smirnova E.S. (2015). Preparation and investigation of ultrafine-grained tungsten carbide with high hardness and fracture toughness. Dokl. Phys..

[52] Chuvil’Deev V.N., Blagoveshchenskii Y.V., Boldin M.S., Sakharov N.V., Nokhrin A.V., Isaeva N.V., Shotin S.V., Lopatin Y.G., Belkin O.A., Smirnovaa E.S. (2015). High-strength ultrafine-grained tungsten-carbide-based materials obtained by spark plasma sintering. Tech. Phys. Lett..

[53] Ghasali E., Ebadzadeh T., Alizadeh M., Razavi M. (2018). Mechanical and microstructural properties of WC-based cermets: A comparative study on the effect of Ni and Mo binder phases. Ceram. Int..

[54] Panov V.S. (2015). Nanostructured sintered WC–Co hard metals (review). Powder Metall. Met. Ceram..

[55] Papynov E.K., Shichalin O.O., Mironenko A.Y., Ryakov A.V., Manakov I.V., Makhrov P.V., Buravlev I.Y., Tananaev I.G., Avramenko V.A., Sergienko V.I. (2018). Synthesis of high-density pellets of uranium dioxide by spark plasma sintering in dies of different types. Radiochemistry.

[56] Papynov E.K., Shichalin O.O., Buravlev I.Y., Portnyagin A.S., Belov A.A., Maiorov V.Y., Skurikhina Y.E., Merkulov E.B., Glavinskaya V.O., Nomerovskii A.D. (2020). Reactive spark plasma synthesis of porous bioceramic wollastonite. Russ. J. Inorg. Chem..

[57] Blagoveshchenskiy Y.V., Isayeva N.V., Blagoveshchenskaya N.V., Melnik Y.I., Chuvildeyev V.N., Nokhrin A.V., Sakharov N.V., Boldin M.S., Smirnov Y.S., Shotin S.V. (2015). Methods of compacting nanostructured tungsten–cobalt alloys from Nanopowders obtained by plasma chemical synthesis. Inorg. Mater. Appl. Res..

[58] Lantcev E., Nokhrin A., Malekhonova N., Boldin M., Chuvil’deev V., Blagoveshchenskiy Y., Isaeva N., Andreev P., Smetanina K., Murashov A. (2021). A study of the impact of graphite on the kinetics of SPS in nano- and submicron WC-10%Co powder compositions. Ceramics.

[59] Ghasali E., Orooji Y., Tahamtan H., Asadian K., Alizadeh M., Ebadzadeh T. (2020). The effects of metallic additives on the microstructure and mechanical properties of WC–Co cermets prepared by microwave sintering. Ceram. Int..

[60] Lantsev E., Malekhonova N., Nokhrin A., Chuvil’deev V., Boldin M., Blagoveshchenskiy Y., Andreev P., Smetanina K., Isaeva N., Shotin S. (2021). Influence of oxygen on densification kinetics of WC nanopowders during SPS. Ceram. Int..

[61] Buravlev I.Y., Shichalin O.O., Papynov E.K., Golub A.V., Gridasova E.A., Buravleva A.A., Yagofarov V.Y., Dvornik M.I., Fedorets A.N., Reva V.P. (2021). WC-5TiC-10Co hard metal alloy fabrication via mechanochemical and SPS techniques. Int. J. Refract. Met. Hard Mater..

[62] Papynov E.K., Shichalin O.O., Apanasevich V.I., Portnyagin A.S., Yu M.V., Yu B.I., Merkulov E.B., Kaidalova T.A., Modin E.B., Afonin I.S. (2020). Sol-gel (template) synthesis of osteoplastic CaSiO_3_/HAp powder biocomposite: “In vitro” and “in vivo” biocompatibility assessment. Powder Technol..

[63] Yarusova S.B., Shichalin O.O., Belov A.A., Azon S.A., Buravlev I.Y., Golub A.V., Mayorov V.Y., Gerasimenko A.V., Papynov E.K., Ivanets A.I. (2022). Synthesis of amorphous KAlSi_3_O_8_ for cesium radionuclide immobilization into solid matrices using spark plasma sintering technique. Ceram. Int..

[64] Papynov E.K.K., Mayorov V.Y.Y., Portnyagin A.S.S., Shichalin O.O.O., Kobylyakov S.P., Kaidalova T.A.A., Nepomnyashiy A.V.V., Sokol׳nitskaya T.A., Zub Y.L.L., Avramenko V.A.A. (2015). Application of carbonaceous template for porous structure control of ceramic composites based on synthetic wollastonite obtained via Spark Plasma Sintering. Ceram. Int..

[65] Papynov E.K., Portnyagin A.S., Modin E.B., Mayorov V.Y., Shichalin O.O., Golikov A.P., Pechnikov V.S., Gridasova E.A., Tananaev I.G., Avramenko V.A. (2018). A complex approach to assessing porous structure of structured ceramics obtained by SPS technique. Mater. Charact..

[66] Shapkin N.P., Papynov E.K., Shichalin O.O., Buravlev I.Y., Simonenko E.P., Simonenko N.P., Zavjalov A.P., Belov A.A., Portnyagin A.S., Gerasimenko A.V. (2021). Spark plasma sintering-reactive synthesis of SiC and SiC–HfB_2_ ceramics based on natural renewable raw materials. Russ. J. Inorg. Chem..

[67] Papynov E.K., Shichalin O.O., Buravlev I.Y., Belov A.A., Portnyagin A.S., Fedorets A.N., Azarova Y.A., Tananaev I.G., Sergienko V.I. (2020). Spark plasma sintering-reactive synthesis of SrWO_4_ ceramic matrices for 90Sr immobilization. Vacuum.

[68] Papynov E.K., Belov A.A., Shichalin O.O., Buravlev I.Y., Azon S.A., Gridasova E.A., Parotkina Y.A., Yagofarov V.Y., Drankov A.N., Golub A.V. (2021). Synthesis of perovskite-like SrTiO_3_ ceramics for radioactive strontium immobilization by spark plasma sintering-reactive synthesis. Russ. J. Inorg. Chem..

[69] Papynov E.K., Shichalin O.O., Belov A.A., Buravlev I.Y., Portnyagin A.S., Azon S.A., Shlyk D.K., Buravleva A.A., Parot’kina Y.A., Nepomnyushchaya V.A. (2021). Synthesis of mineral-like SrWO_4_ ceramics with the scheelite structure and a radioisotope product based on it. Russ. J. Inorg. Chem..

[70] Simonenko E.P., Simonenko N.P., Gordeev A.N., Kolesnikov A.F., Papynov E.K., Shichalin O.O., Tal’skikh K.Y., Gridasova E.A., Avramenko V.A., Sevastyanov V.G. (2018). Impact of a supersonic dissociated air flow on the surface of HfB_2_–30 vol % SiC UHTC produced by the Sol–Gel method. Russ. J. Inorg. Chem..

[71] Simonenko E.P., Simonenko N.P., Gordeev A.N., Papynov E.K., Shichalin O.O., Kolesnikov A.F., Avramenko V.A., Sevastyanov V.G., Kuznetsov N.T. (2018). Study of the thermal behavior of wedge-shaped samples of HfB_2_–45 vol % SiC ultra-high-temperature composite in a high-enthalpy air flow. Russ. J. Inorg. Chem..

[72] Simonenko N.P., Simonenko E.P., Mokrushin A.S., Popov V.S., Vasiliev A.A., Sevastyanov V.G., Kuznetsov N.T. (2017). Thin films of the composition 8% Y_2_O_3_–92% ZrO_2_ (8YSZ) as gas-sensing materials for oxygen detection. Russ. J. Inorg. Chem..

[73] Simonenko T.L., Kalinina M.V., Simonenko N.P., Simonenko E.P., Glumov O.V., Mel’nikova N.A., Murin I.V., Shichalin O.O., Papynov E.K., Shilova O.A. (2019). Synthesis of BaCe_0.9x_ZrxY_0.1_O_3_ nanopowders and the study of proton conductors fabricated on their basis by low-temperature spark plasma sintering. Int. J. Hydrogen Energy.

[74] Papynov E.K., Shichalin O.O., Mayorov V.Y., Kuryavyi V.G., Kaidalova T.A. (2019). SPS technique for ionizing radiation source fabrication based on dense cesium-containing core. J. Hazard. Mater..

[75] Kosyanov D.Y., Yavetskiy R.P., Vorona I.O., Shichalin O.O., Papynov E.K., Vornovskikh A.A., Kuryavyi V.G., Vovna V.I., Golokhvast K.S., Tolmachev A.V. (2017). Transparent 4 at% Nd^3+^:Y_3_Al_5_O_12_ ceramic by reactive spark plasma sintering. AIP Conference Proceedings.

[76] Golovkina L.S., Orlova A.I., Chuvil’deev V.N., Boldin M.S., Lantcev E.A., Nokhrin A.V., Sakharov N.V., Zelenov A.Y. (2018). Spark plasma sintering of high-density fine-grained Y_2.5_Nd_0.5_Al_5_O_12+_SiC composite ceramics. Mater. Res. Bull..

[77] Zavjalov A.P., Nikiforov P.A., Kosyanov D.Y., Zakharenko A.M., Trukhin V.O., Talskikh K.Y., Shichalin O.O., Papynov E.K. (2020). Phase formation and densification peculiarities of Hf–C–N solid solution ceramics during reactive spark plasma sintering. Adv. Eng. Mater..

[78] Kosyanov D.Y., Liu X., Vornovskikh A.A., Kosianova A.A., Zakharenko A.M., Zavjalov A.P., Shichalin O.O., Mayorov V.Y., Kuryavyi V.G., Qian X. (2021). Al_2_O_3_–Ce:YAG and Al_2_O_3_–Ce:(Y,Gd)AG composite ceramics for high brightness lighting: Effect of microstructure. Mater. Charact..

[79] Mendelson M.I. (1969). Average grain size in polycrystalline ceramics. J. Am. Ceram. Soc..

[80] Rahaman M.N. (2003). Ceramic Processing and Sintering.

[81] Liu X., Song X., Zhao S., Zhang J. (2010). Spark plasma sintering densification mechanism for cemented carbides with different WC particle sizes. J. Am. Ceram. Soc..

